# Interaction between **γ**-Aminobutyric Acid A Receptor Genes: New Evidence in Migraine Susceptibility

**DOI:** 10.1371/journal.pone.0074087

**Published:** 2013-09-05

**Authors:** Marlene Quintas, João Luís Neto, José Pereira-Monteiro, José Barros, Jorge Sequeiros, Alda Sousa, Isabel Alonso, Carolina Lemos

**Affiliations:** 1 UnIGENe IBMC – Instituto de Biologia Molecular Celular, Universidade do Porto, Porto, Portugal; 2 ICBAS, Instituto de Ciências Biomédicas Abel Salazar, Universidade do Porto, Porto, Portugal; 3 Serviço de Neurologia, CHP-HSA, Centro Hospitalar do Porto, Hospital de Santo António. Abel Salazar, Porto, Portugal; National Cancer Institute, National Institutes of Health, United States of America

## Abstract

Migraine is a common neurological episodic disorder with a female-to-male prevalence 3- to 4-fold higher, suggesting a possible X-linked genetic component. Our aims were to assess the role of common variants of gamma-aminobutyric acid A receptor (GABA_A_R) genes, located in the X-chromosome, in migraine susceptibility and the possible interaction between them. An association study with 188 unrelated cases and 286 migraine-free controls age- and ethnic matched was performed. Twenty-three tagging SNPs were selected in three genes (*GABRE, GABRA3 and GABRQ*). Allelic, genotypic and haplotypic frequencies were compared between cases and controls. We also focused on gene-gene interactions. The AT genotype of rs3810651 of *GABRQ* gene was associated with an increased risk for migraine (OR: 4.07; 95% CI: 1.71-9.73, p=0.002), while the CT genotype of rs3902802 (OR: 0.41; 95% CI: 0.21-0.78, p=0.006) and GA genotype of rs2131190 of *GABRA3* gene (OR: 0.53; 95% CI: 0.32-0.88, p=0.013) seem to be protective factors. All associations were found in the female group and maintained significance after Bonferroni correction. We also found three nominal associations in the allelic analyses although there were no significant results in the haplotypic analyses. Strikingly, we found strong interactions between six SNPs encoding for different subunits of GABA_A_R, all significant after permutation correction. To our knowledge, we show for the first time, the putative involvement of polymorphisms in GABA_A_R genes in migraine susceptibility and more importantly we unraveled a role for novel gene-gene interactions opening new perspectives for the development of more effective treatments.

## Introduction

Migraine is a common and often debilitating neurological disorder. The knowledge of its underlying pathophysiology is still limited, although it is considered as a peculiar response of the central nervous system (CNS) to a variety of environmental and genetic components [1,2]. Mutations or polymorphisms in genes involved in ion channel and neurotransmitter pathways, vascular functions and hormonal mechanisms are considered key factors for migraine susceptibility [3]. Experimental pharmacology and human genetic data support a model for migraine based on a neuronal hyperexcitability and activation of the trigeminovascular system [4,5].

Migraine prevalence is in general, 3- to 4-fold higher among women [6] and several hypotheses have been raised for this female predominance [7,8]. We have also shown in the Portuguese population that gender is a critical risk factor for migraine and a gender-biased transmission is observed [9]. This can be explained by a migraine susceptibility locus on the X-chromosome [10], Xq24-28, which was identified in an Australian study in two large families [11].

Gamma-aminobutyric acid (GABA) is the main inhibitory neurotransmitter in the CNS, being released in approximately one third of all synapses [12,13]. . GABA plays an important role in neuronal proliferation, migration, differentiation and in the regulation of pain, perception and memory pathways [14,15]. Consequently, an impaired GABAergic transmission has been implicated in a wide range of neurological and psychiatric disorders [16–18]. GABA agonists have also been used as prophylactic drugs for migraine [19–21]. GABA binds to different receptors, including the GABA-A receptor (GABA_A_R), a pentameric complex of multiple subunits – α1-α6, β1-β3, γ1-γ3, δ, ε, π and θ – encoded by several different genes [22]. GABA_A_Rs are ionotropic transmembrane chloride channels, which mediate fast inhibitory neurotransmission. Alterations in their trafficking, synaptic accumulation or function have a crucial role in the regulation of neuronal excitability [23].

In the Xq24-28 locus there is a cluster of GABA_A_R subunit genes, *GABRE*, *GABRA3* and *GABRQ* encoding ε, α and θ subunits. A previous study has investigated the association between the *GABRQ* and *GABRE* genes and migraine susceptibility, but no associations were found with the SNPs tested [24].

This study aimed (i) to unravel the role of *GABRE, GABRA3* and *GABRQ* genes as migraine susceptibility factors through an association study approach in a Portuguese sample (ii) to explore the impact of variants in gender-related migraine susceptibility and (iii) to assess a possible interaction between GABA_A_R genes in migraine. In this study we found an association between GABA_A_R genes and migraine susceptibility and importantly we disentangled three gene-gene interactions between these genes, which can be relevant to understand the disease’s pathways.

## Material and Methods

### Subjects

A sample of 188 unrelated migraine patients (153 females and 35 males) from the out-patient neurology clinic, at Hospital de Santo António (HSA), Porto, was sequentially enrolled in this study. The sample included 111 migraine without aura (MO) and 77 with migraine with aura (MA). Patients with familial and sporadic hemiplegic migraine were excluded.

Control subjects (n=286; 217 females and 69 males), with no personal history of migraine, were ascertained among healthy blood donors and from the Department of Obstetrics and Gynecology. Controls were from the same ethnic and geographical origin (north of Portugal) as cases, and were age-matched to these. A diagnostic interview was performed both in cases and controls, based on the operational criteria of the International Headache Society (IHS), using the same structured questionnaire.

Samples were ascertained between 1999 and 2004 and thus the first edition of these criteria (ICHD-I) was used; as no major differences in common migraine diagnosis were introduced by the 2004 IHS criteria revision we did not expected changes in this cohort clinical diagnosis. Nevertheless, we have revised the clinical diagnosis of all patients applying the second edition (ICHD-II) and no differences in patients’ diagnosis were found (data not shown). All subjects provided a written informed consent prior to participation and the project was approved by the Ethics Committee of HSA.

### SNP selection and genotyping

Genomic DNA was extracted from peripheral blood leukocytes, using the standard salting out method [25] or from saliva, using ORAGENE kits and DNA extraction was performed according to the manufacturer’s instructions (DNA Genotek, Inc.).

Single-nucleotide polymorphisms (SNPs) were selected based on a data dump from the International HapMap Project; tagging SNPs were selected using Haploview 4.1, using r^2^ as a measure of linkage disequilibrium (LD) at a threshold of 0.80, with a minor allele frequency (MAF) ≥ 0.10, by an aggressive tagging approach (a multimarker method) [26]. Twenty-three tagging SNPs (Table **1**) were selected in order to achieve the maximum genotypic information and multiplexed into 2 assays. The method chosen for genotyping SNPs variants was SNaPshot (Applied Biosystems).

**Table 1:** Tagging SNPs selected for each gene.

	**Gene**		
	*GABRA3*	*GABRQ*	*GABRE*
**Tagging SNPs**	rs10218364		rs2266858
	rs2131190		rs1158605
	rs5925155		rs2256882
	rs6627595	rs5925196	rs5925077
	rs10482215	rs5924752	rs1061418
	rs2201169	rs3810651	rs1139916
	rs6627588	rs5924753	rs5970170
	rs5970223		rs1003794
	rs7391474		rs2266856
	rs3902802		

PCR primers and SNaPshot SBE primer sequences for the 23 variants studied are presented as supplementary data (Table S1 a) and b)).

Multiplex PCR amplification was performed in a final volume of 10µL containing 5.0µL of multiplex PCR Master Mix (Qiagen) enzyme, 1.0µL of a suitable amplification primer mix, 1.0µL of DNA (10ng/µL), and 3.0µL of water. Initially the primer mix contained the forward and reverse amplification primers of all SNPs, at a concentration of 2µM. In the optimization phase, when necessary, adjustments to the concentrations were performed in order to obtain balanced peaks. The cycling conditions used were: an initial denaturing at 95^°^C for 15 min, followed by 30 cycles of 30 sec at 94^°^C, 90 sec at 55^°^C and 90 sec at 72^°^C, followed by a final extension step of 10 min at 72^°^C. For PCR product purification, incubation with ExoSAP-IT was performed, according to the manufacturer’s instructions. This enzymatic purification was performed in two steps: first, 15 min at 37^°^C, to remove remaining primers and nucleotides and, second, 15 min at 85^°^C to inactivate the enzymes.

The SNaPshot extension reactions were carried out in a final volume of 5.0µL, containing 1.5µL of the purified PCR product, 1.0µL of SNaPshot Multiplex Mix (Applied Biosystems) having the fluorescent ddNTPs, 1.0µL of an SBE-primer mix and 1.5µL of water.

After primer extension, the unincorporated fluorescently labeled ddNTPs were removed by adding 1 µL of SAP (USB Corporation) and subjected to an incubation at 37^°^C for 90 min, followed by 15 min at 85^°^C for enzyme inactivation. Detection was carried out using 1.0 µl of SNaPshot products mixed with 8.85 µl of formamide and 0.15 µl of GeneScan-120 LIZ size standard (Applied Biosystems). Fragments were separated by capillary electrophoresis on an automated sequencer (ABI-PRISM 3130 XL Genetic Analyzer - Applied Biosystems) and analyzed with GeneMapper analysis software version 4.0 (Applied Biosystems). To confirm particular genotypes some SNPs were additionally genotyped by automated-sequencing or restriction fragment length polymorphism (RFLP) analysis.

### Statistical analysis

Power to detect association was estimated with the Genetic Power Calculator (http://pngu.mgh.harvard.edu/~purcell/gpc/), assuming a codominant genetic model with a high-risk allele frequency of 0.1, a relative risk for a homozygous genotype of 2.25 and 1.5 in heterozygosity. Analysis of Hardy-Weinberg equilibrium (H–W) was performed using SNPator software [27].

To compare allele frequencies between cases and controls, the SNPator software was also used. A chi-square (χ^2^) test was performed and odds ratio (OR) were estimated with 95% confidence intervals (CI). The significant level was set at α=0.05.

A backward-stepwise multivariable-logistic regression was performed (with the most frequent homozygote as the reference group), to evaluate association between SNP’s genotypes and the occurrence of migraine; this was only done in the female group. To correct for multiple comparisons a Bonferroni correction was performed, taking into account α=0.016 (considering that the SNPs for each of the three genes were simultaneously analyzed in the logistic regression). These analyses were performed with PASW Statistics v 18.0 software.

Significant results found were further evaluated using multifactor dimensionality reduction (MDR) software (version 2.0). MDR is a non-parametric and genetic model-free approach that can identify SNPs involved in disease susceptibility [28]. A single-locus analysis for main effects was conducted with MDR and we also performed a gene-gene interaction analysis [29]. We used a ten-fold cross-validation to avoid false-positives [30]. The significant results obtained were corrected for multiple testing using the permutation test implemented on the MDR Permutation Tool (version 1) [31].

Haplotype frequencies were compared between cases and controls using Haploview 4.1, with default settings. Frequencies of haplotypes analyzed were above 1% according to the Haploview threshold.

To correct for multiple comparisons, regarding the estimation of allelic and haplotypic frequencies, permutations tests were performed in Haploview using 10,000 permutations.

For the prediction of putative functional roles of the associated SNPs, we employed the SNP Function Prediction (FuncPred) [32] bioinformatic tool (http://snpinfo.niehs.nih.gov/snpfunc.htm). This analysis also predicted possible functions for SNPs in LD (r^2^ ≥ 0.8) in the European population to the queried associated SNPs. PROVEAN [33], SIFT [34], and Polyphen-2 [35] were used to evaluate the impact of SNPs causing non-synonymous amino-acid modifications.

## Results

The demographic data of our sample are presented in Table **2**. We obtained a case:control ratio of 1:1.5 and no significant differences were found regarding gender between patients and controls (p>0.05). The power of our sample to detect association was 64% (for a nominal α=0.05). A prevalence of 16% for migraine had been previously estimated in the Portuguese population [36]. Cases and controls were in Hardy-Weinberg equilibrium for all the tagging SNPs selected. The correlation between GABA_A_R SNPs was small, denoting the weak LD between them (Figure S1). Taking into account that GABA_A_R genes are located in the X-chromosome our analysis was stratified by gender and genotypic analyses were only performed in the female group.

**Table 2:** Demographic and clinical data of patients with migraine and controls.

**Characteristics**	**Patients with migraine (n=188)**	**Controls (n=286)**
Gender, F/M	153/35	217/69
Age at observation, mean (SD), y	36.14 (12.84)	36.42 (12.35)
Age at onset, mean (SD), y	11.67 (8.15)	n/a
Family history of migraine, %	87	n/a

### Allelic frequencies

Regarding allelic frequencies we found an enrichment of the T allele of rs5925077 of *GABRE* gene (OR: 2.13; 95% CI: 1.16-3.90; *p*=0.014) and of the C allele of rs2201169 of *GABRA3* gene (OR: 2.26; 95% CI: 1.05-4.85; *p*=0.033) among the male patients; however the results did not retained statistical significance after permutation-based correction. Additionally, in the female group we have found an increased risk conferred by the G allele of *GABRA3* rs2131190, for migraine susceptibility (OR=1.53, 95%CI: 1.01-2.30; *p* = 0.043), but that did not remain significant after multiple testing correction. Allelic frequencies for the 23 studied variants are presented as supplementary material (Table S2).

### Genotypic analyses

Statistically significant results from the backward stepwise multivariable logistic regressions performed are presented in Table **3**. The AT genotype of rs3810651 of *GABRQ* gene showed an increased risk for migraine (OR: 4.07; 95% CI: 1.71-9.73, p=0.002), still significant after Bonferroni correction. On the other hand the CT genotype of rs3902802 and the GA genotype of rs2131190 of *GABRA3* gene were associated with a decreased risk for migraine (CT-OR: 0.41; 95% CI: 0.21-0.78, p=0.006; GA-OR: 0.53; 95% CI: 0.32-0.88, p=0.013), that also remained significant after Bonferroni correction.

**Table 3:** Results from multivariable logistic regression found in the female group.

**Gene**	**SNP**	**Genotypic frequencies N. (%)**		**OR (95% C.I.)**	**p**
		**Cases**	**Controls**		
**GABRA3**	**rs2131190**				0.018
	GG (ref)	115 (75.2)	143 (65.9)	1	
	AG	36 (23.5)	67 (30.9)	0.53 (0.32-0.88)	0.013*
	AA	2 (1.3)	7 (3.2)	0.25 (0.05-1.29)	0.10
	**rs7391474**				0.008
	TT (ref)	88 (57.5)	143 (65.9)	1	
	GT	59 (38.6)	65 (30.0)	1.42 (0.42-4.75)	0.57
	GG	6 (3.9)	9 (4.1)	0.61 (0.18-2.07)	0.43
	**rs3902802**				0.02
	TT (ref)	125 (81.7)	168 (77.4)	1	
	CT	25 (16.3)	46 (21.1)	0.41 (0.21-0.78)	0.006*
	CC	3 (2)	3 (1.4)	1.12 (0.20-6.31)	0.90
**GABRQ**	**rs5924753**				0.08
	TT (ref)	42 (27.5)	63 (29.0)	1	
	CT	74 (48.4)	105 (48.4)	0.35 (0.14-0.88)	0.03
	CC	37 (24.2)	49 (22.6)	0.35 (0.11-1.17)	0.09
	**rs3810651**				0.01
	AA (ref)	46 (30.0)	91 (41.9)	1	
	AT	78 (51.0)	88 (40.6)	4.07 (1.71-9.73)	0.002*
	TT	29 (19.0)	38 (17.5)	3.29 (0.99-10.94)	0.05
	**rs5925196**				0.09
	TT (ref)	109 (71.3)	142 (65.4)	1	
	AT	36 (23.5)	70 (32.2)	0.69 (0.42-1.15)	0.15
	AA	8 (5.2)	5 (2.3)	2.32 (0.69-7.74)	0.17
**GABRE**	**rs2256882**				0.05
	AA (ref)	125 (81.7)	165 (76.0)	1	
	AG	22 (14.4)	51 (23.5)	0.08 (0.01-0.72)	0.02
	GG	6 (3.9)	1 (4.6)	0.12 (0.02-1.03)	0.05

(ref) indicates the genotype reference category.

OR − odds ratio; CI − confidence interval

After Bonferroni correction, significance level was set to 0.016; * Significant values.

Using MDR we performed a single-locus analysis and found that the best model for rs3810651 showed a testing balance accuracy (TBA) of 0.54 and a cross validation (CVC) of 10/10 in the female group. After permutation testing, this model was still significant (p=0.025), which confirmed the logistic regression analysis results and the importance of this SNP in migraine susceptibility.

We have also performed a haplotype-based analysis, but no differences were found between cases and controls for any of the haplotypes evaluated (data not shown).

### Gene-gene interaction

**Figure 1:**
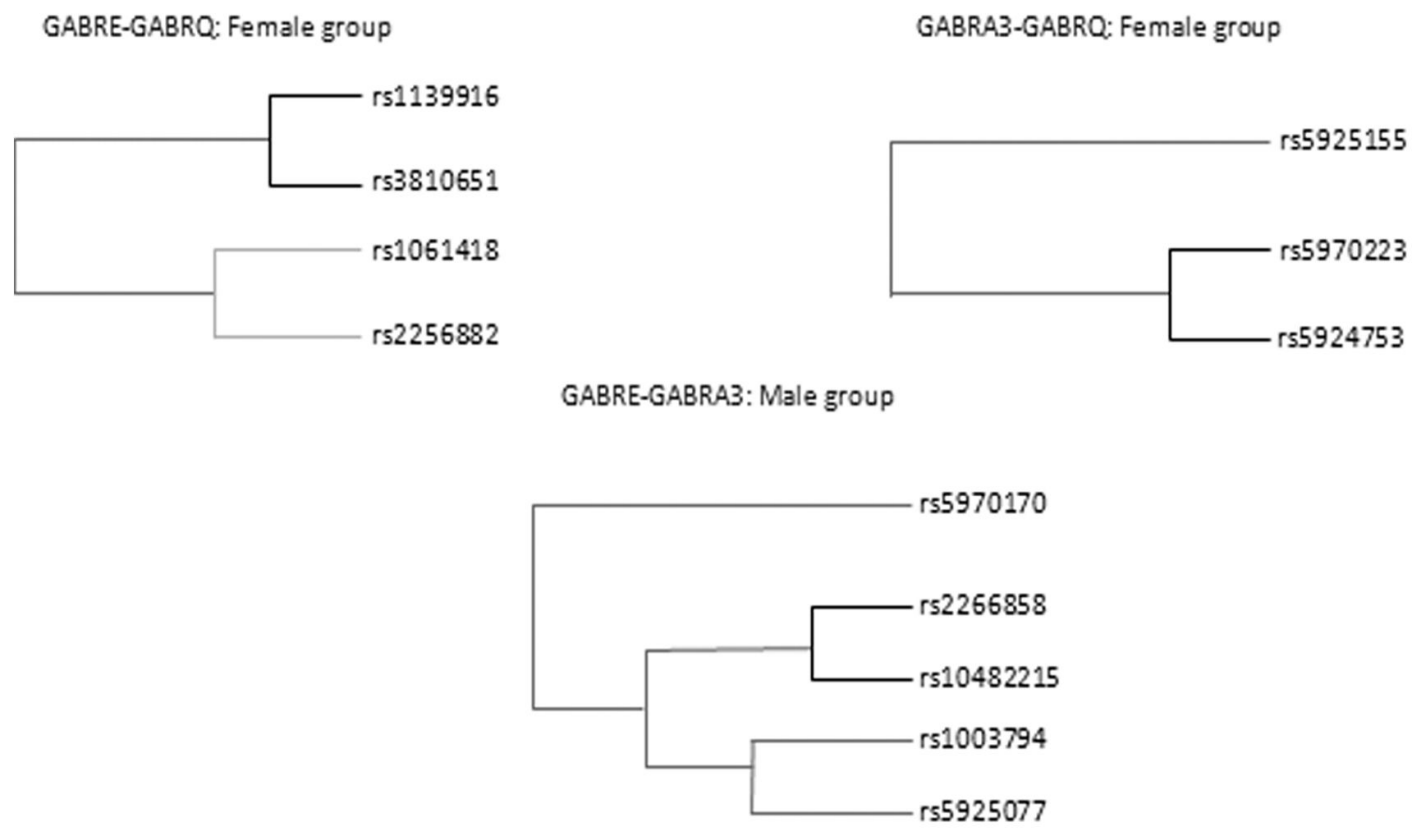
Gene-gene interaction dendrograms showing significant results and a strong interaction effect between SNPs of *GABRE*-*GABRQ* and *GABRA3*-*GABRQ* and *GABRE*-*GABRA3* (darker lines suggest a synergistic relationship: the shorter the lines, the stronger the interaction).

Results from the multilocus MDR analyses are summarized in Figure **1**. We observed consistency in the CVC measures throughout all models (10/10). Based on CVC, TBA and permutation p-values, we found strong and significant interactions. In the female group we observed two significant models of interaction – one between rs5970223 * rs5924753 that showed a TBA of 0.62 (p=0.0074), while the other interaction, rs3810651 * rs1139916, showed a TBA of 0.62 (p=0.0045). In the male group an interaction between rs10482215 * rs2266858 was found with a TBA of 0.70 (p=0.020). To reinforce these results we also performed a backward stepwise multivariable logistic regression including the interaction terms and the results were consistent between the two methods (data not shown).

### Functional in silico analysis

To explore the functional impact of the SNPs found to be associated with migraine we have performed a bioinformatics analysis. From this analysis important results have emerged involving the three migraine associated SNPs. The nucleotide variation of rs5925077 was predicted to alter transcription factors’ binding (TFB) sites with a higher number of TFB sites when the T allele was present. Also, we found that rs1139916 was predicted to result in alterations in the recognition sites for splicing regulatory factors. Specifically, the C variant was predicted to modify exonic splicing enhancer (ESE) sites. Additionally, we found that rs1139916 and rs3810651 are non-synonymous SNPs resulting in alterations in the amino acid sequence of the receptor. rs3810651 is responsible for a phenylalanine to isoleucine (Phe478Ile) change in the θ subunit (*GABRQ*), while rs1139916 causes a serine to alanine (Ser102Ala) substitution in the ε subunit (*GABRE*). The FuncPred analysis included a Polyphen [37] prediction where both mutations were classified as benign. To be additionally thorough, the effect of these missense mutations was also evaluated using other tools. PROVEAN scored the two substitutions as neutral, SIFT - using either homologues or orthologues for the protein alignment - predicted that both variations should be tolerated, and in Polyphen-2 (a newer development of Polyphen), rs3810651 was still categorized as benign, but rs1139916 was now classified as possibly damaging. 

## Discussion

While many unanswered questions remain around migraine pathophysiology, some evidence point to the involvement of GABA and it receptors in this process [38]. Consequently GABA_A_R genes are seen as potential candidates for migraine therapies.

We analyzed the distribution of allelic, genotypic and haplotypic frequencies of twenty-three SNPs localized in the *GABRE*, *GABRA3* and *GABRQ* genes to explore the role of these genes in migraine susceptibility using a case-control approach.

Our findings confirm the involvement of GABA_A_R genes in migraine’s susceptibility. In our sample we found three nominal significant allelic associations, two in the male group (rs5925077 and rs2131190) and the other in the female group (rs2201169); however none of them remained significant after permutation-based correction.

Regarding the genotypic analyses a set of interesting results reinforced the role of two genes (*GABRA3* and *GABRQ*) in migraine. The CT and the AG genotypes of rs3902802 and rs2131190, were associated with a decrease in the risk for migraine, emerging as possible protective factors, while the AT genotype of rs3810651 reflected an increased risk for migraine. All associations resisted to Bonferroni correction. These data suggest that multiple alleles play a role in migraine susceptibility and that the presence of risk alleles and/or absence of protective variants may influence the onset of the disease symptoms.

All genes investigated in this study have a chromosomal location on Xq28, supporting the implication of this genomic region in migraine pathogenesis. Also, it is imperative to note that the differences observed between genders cannot be solely explained by the influence of GABA_A_R genes. Hormones, namely estrogens - involved in pain pathways - and progesterone which might decrease the occurrence of migraine [39], should also be taken into account. Another noteworthy aspect is that men might be protected by testosterone, since this hormone seems to have a protective role in pain development [39]. These facts may also explain the differential gender ratio found for this disorder and it would be important to assess to what extent our results reflect a true gender-specific effect versus a low representation of males in our sample.

We also employed a MDR analysis for the detection of gene–gene interactions, which is a useful data reduction method for detecting multilocus genotype combinations that predict risk for complex diseases [40]. It combines cross-validation and permutation testing to minimize false positive results [28]. With the MDR analysis we confirmed the evidence of gene-gene interaction between GABA_A_R genes in migraine susceptibility. Previously, we had already found a strong gene-gene interaction in migraine susceptibility using this same method [41]. In complex diseases, interaction is a ubiquitous phenomenon that contributes to the development of the disorder [42], so it is expected that multiple genes, each with a weak or moderate effect, will have a greater joint contribution to disease risk than a single gene [43].

In order to gain some insight regarding the putative functional consequences of the SNPs found to be associated with migraine, we have performed a bioinformatic analysis for two SNPs that result in alterations in the protein coding sequence (rs3810651 and rs1139916). The majority of the software’s used predict that these variants do not have an impact on protein function, except Polyphen-2 that classifies rs1139916 as possibly damaging. Although very useful to infer the effect of non-coding SNPs on protein function, interpretation of these software predictions should be cautious, as they mainly rely only on evolutionary conservation of a given protein position. In particular, for the variants studied here, data on the predicted topological organization of the θ and ε subunits can give some clues on the impact of these variants. The Phe478Ile variant (rs3810651) is located in the major intracellular loop and we can hypothesize that this substitution may result in altered protein interactions [44]. Regarding the Ser102Ala (rs1139916) it is located in the first extracellular domain of the subunit and this alteration could affect the binding of extracellular ligands. In both cases the functional properties of GABA_A_R may be affected by these variants and thus contribute to disease. Despite these functionally relevant locations, experimental validation is required to confirm these hypotheses.

A connection between the GABA receptor genes and migraine has been investigated over the last decade, although, until now, few positive associations were found. A linkage analysis in 10 families with migraine with aura used markers from the 15q11-q13 genomic region and hypothesized a possible relation between GABA_A_R dysfunction and migraine [45]. Two association studies exploring that hypothesis did not find any convincing evidence [46,47]. Also, Chen et al. investigated the role of one SNP in *GABRG2* gene located on chromosome 5q31.1-q33.1, but no significant differences in allele frequencies were found [48]. An Australian study [24] focused on candidate genes in the X-chromosome (*GABRE, GABRQ*) and their involvement in migraine but no association was found with the SNPs tested. In our study we analyzed two SNPs also studied by the Australian group and for one of these SNPs (rs3810651) we found a significant association with migraine. The difference between our results and those found in the Australian population may be due to allele frequency variation across populations and could also be influenced by gene-gene and/or gene-environment interactions.

Our study pinpoints the GABA_A_R genes as factors that could modulate the migraine liability, but a comprehensive vision of the molecular basis of disease is still lacking. Plummer et al. [49] performed a study aiming to assess the GABA_A_R genes’ expression profile in migraineurs and controls. The study demonstrated that *GABRA3* was significantly down regulated in female patients compared with controls which could result in a decrease of GABA_A_R activation. These results reinforce our findings and it would be important to assess the gene expression, taking into account the patients’ genotypes. Also, would be important to identify MA and MO specific risk factors in a larger cohort.

Despite the fact that our sample is not very large, a special care in obtaining a high case to control ratio to increase power was taken into account. Moreover, cases and controls were matched for age at observation and geographic region and corrections for multiple testing (Bonferroni and permutation-based corrections) were applied.

In conclusion, we believe that our study provides important insights into the role of GABA_A_R genes in migraine susceptibility and in gender-liability differences. As the first study to present positive data on the impact of variants in the GABA_A_R genes, our results are suggestive for the involvement of these genes in migraine susceptibility. Nevertheless, further investigation is necessary, in particular the replication of these findings in other populations and experimental functional assays that confirm the impact of these variants in normal channel function. The multigenic nature of migraine makes the identification of disease-related genes challenging and gene-gene interactions may be fundamental mechanisms for the development of therapies for complex diseases. Further investigation is necessary taking into to account gene-gene interaction, as well as, epigenetic mechanisms to increase knowledge on the genetic basis of this disease and contribute to the development of more effective treatments.

## Supporting Information

Figure S1 A. Genomic organization of the GABA_A_R genes cluster in Xq24-28 with the size of each gene and the distance between them. B. LD plots showing both D’ and R^2^ are shown. These plots are based on genotype data from our control sample for the 23 variants analyzed in this study. Noteworthy, the two plots show a correlation between rs3810651 and rs5924753 in our population contrarily to the HapMap prevision.
(TIF)

Table S1 PCR primers and SNaPshot SBE primer sequences.
(DOCX)

Table S2 Allele frequencies of SNPs studied in patients with migraine and controls
(XLSX)
