# Supplementary figures and images for: Interaction between γ-Aminobutyric Acid A Receptor Genes: New Evidence in Migraine Susceptibility
Source: PLoS One. 2013 Sep 5;8(9):e74087. doi: 10.1371/journal.pone.0074087 (PMC3764027; doi:10.1371/journal.pone.0074087)

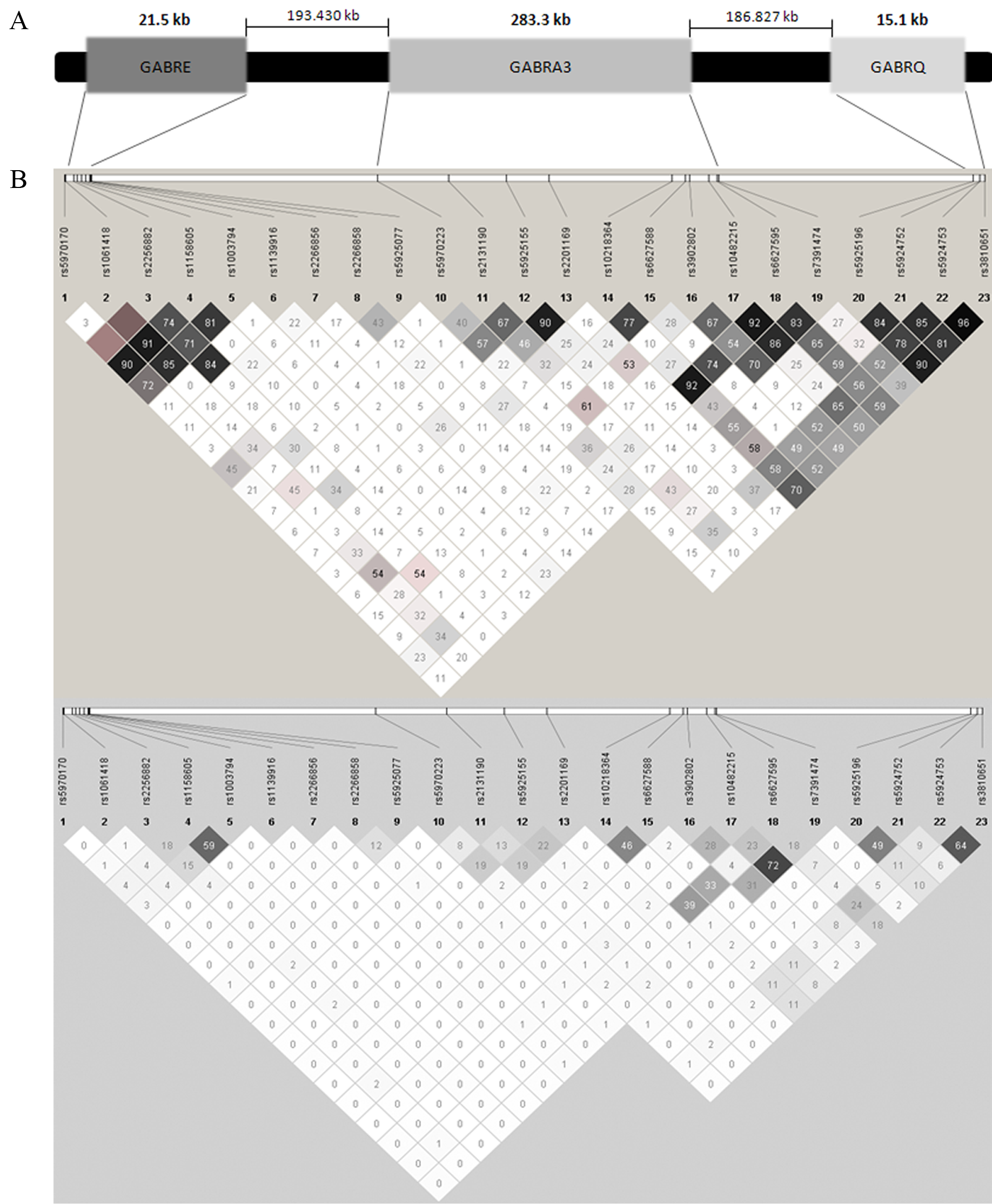

Supplement: Figure S1 — A. Genomic organization of the GABAAR genes cluster in Xq24-28 with the size of each gene and the distance between them. B. LD plots showing both D’ and R2 are shown. These plots are based on genotype data from our control sample for the 23 variants analyzed in this study. Noteworthy, the two plots show a correlation between rs3810651 and rs5924753 in our population contrarily to the HapMap prevision. (TIF) [file pone.0074087.s001.tif]
