# Supplementary material for: Interaction between γ-Aminobutyric Acid A Receptor Genes: New Evidence in Migraine Susceptibility
Source: PLoS One. 2013 Sep 5;8(9):e74087. doi: 10.1371/journal.pone.0074087 (PMC3764027; doi:10.1371/journal.pone.0074087)
Supplement: Table S1 — PCR primers and SNaPshot SBE primer sequences. (DOCX) [file pone.0074087.s002.docx]

| **Gene** | **SNP** | **Foward 5` - 3`** | **Reverse 5` - 3`** | **Size of amplicon** |
| --- | --- | --- | --- | --- |
| **GABRE** | rs5970170 | GCAAGTGATAGAACTCCGAC | GTTGCAAAGCCGAGTTTCCT | 82 |
|  | rs1061418 | TTGTCTTTGCTGCCCCTCTC | TAATAAATGGGCCACCTCCC | 90 |
|  | rs2256882 | ACTCACAGCTAGAGAAAGAT | GTCATCCTGGAAGGGAGAAA | 103 |
|  | rs1158605 | CTACTACTCACTTTGTGCTG | GGCAAATGTGACTATGGTCC | 112 |
|  | rs1003794 | TTTAACACCACATGTGAGGG | GTGTTCCATGCTAGGTTTGG | 133 |
|  | rs1139916 | TTGACACTTACCCTCTGCTG | GACCTTCCACAGACTTCTGC | 179 |
|  | rs2266856 | GACTGTTGGTGGTGTGAGAA | CAGGGAGAGGTCTTCTGGTT | 189 |
|  | rs2266858 | AGCAAGCAGCTACACATCCC | GACAAACAAGACACAAGCCC | 102 |
|  | rs5925077 | CAAGACTCCGAGGAGTGCTT | GAGCCCATCCTACAGTGAG | 147 |
| **GABRA 3** | rs5970223 | TTCCCTACTGTCTCTGTCCC | GCTGAAGAGGATGGAGAAAG | 128 |
|  | rs2131190 | ACAGAGAGCTAAACAGACAC | GAGCCCTTAGATTGCTGTGA | 80 |
|  | rs5925155 | AAGAAAGTGATCCTTGTACC | CCTTTGGCTCACCTTGTATT | 118 |
|  | rs2201169 | GAGAAGAGAGGAGAGAGTAG | GTTTGACATGAAGGTCTGTG | 70 |
|  | rs10218364 | AGCACACATCAAACCATTCC | CCGTCATTATCAGAATTCCC | 98 |
|  | rs6627588 | TCCCTGATGTACTTCTTGAA | GCTCCTTTTCATCCTGTAGG | 116 |
|  | rs3902802 | TCATTGTACAAGTGGCATTC | TTTCTGAGTGCCTGTTTAGG | 128 |
|  | rs10482215 | CAACTAAACATACATTGCCC | GTAAGGTACCATTTTGCCC | 182 |
|  | rs6627595 | AGAACAACTCCAAATGCTCA | GGAAAGGAAGCTGACCAATC | 128 |
|  | rs7391474 | AACAAGTTGGAGGTTGAGCC | GTCTGTCTCTCTGGTTTGCT | 104 |
| **GABRQ** | rs5925196 | TCACAGATTCACTCACCTGC | CTTACACACATACTCTTGCA | 86 |
|  | rs5924752 | GTATCTCCTGTGCTCCCTCC | AGAGCATAAACGGTCCTCTG | 193 |
|  | rs5924753 | GGTGGAGAGATAGATAGGTG | CTGGGACTAGGTGGTCTTTA | 80 |
|  | rs3810651 | TCACTTCTCTCTCAGGCCAG | TGTTACCCATGACCATGAAG | 192 |

**Table S1 a): PCR primers and SNaPshot SBE primer sequences**

**Table S1 b): SNaPshot SBE primer sequences**

| **ASSAY 1** | |
| --- | --- |
| rs2266858_GABRE | tCACATCCCCTTCTCTGA |
| rs2131190_GABRA3 | tctgacaaGTGGTAACGTCTAAGACT |
| rs1158605_GABRE | gtgaaagtctgacaatcAGGTCAACCACACATTT |
| rs2256882_GABRE | ccacgtcgtgaaagtctgacaaTCCAGGATGACCATTGATGC |
| rs1061418_GABRE | tgccacgtcgtgaaagtctgacaaCTACCTGGCCCATTCACTGAGT |
| rs1139916_GABRE | aactaggtgccacgtcgtgaaagtctgacaaGGACCAAGGCTGTTGACGG |
| rs5924753_GABRQ | actaaactaggtgccacgtcgtgaaagtctgacaaAGAGATAGATAGGTGATAGATAA |
| rs5970170_GABRE | tgactaaactaggtgccacgtcgtgaaagtctgacaattGGTGGGCACCAATCAATGGCGAG |
| rs5925196_GABRQ | actgactaaactaggtgccacgtcgtgaaagtctgacaacccccCACATCTACATTGATAGGTACA |
| rs2201169_GABRA3 | ccccaactgactaaactaggtgccacgtcgtgaaagtctgacaaggAGGGAAGGACAGGGAGGAAAGGAA |
| rs5924752_GABRQ | ccccccccaactgactaaactaggtgccacgtcgtgaaagtctgacaaCTCCACCATCTTTCTCT |
| rs10218364_GABRA3 | cccccccccccccaactgactaaactaggtgccacgtcgtgaaagtctgacaaggAGGGAAGGACAGGGAGGAAAGGAA |
| **ASSAY 2** | |
| rs1003794_GABRE | ctgacaaGACTGGTTCAGAAAAGAGA |
| rs5925155_GABRA3 | gaaagtctgacaagCTTTCCATACTAAATGCCCC |
| rs5925077_GABRE | tcgtgaaagtctgacaatCTCCAAAAGGAAGACAGGACGCCA |
| rs6627595_GABRA3 | acgtcgtgaaagtctgacaaggggaAGGCTATAAAAAGTGAAGTAC |
| rs10482215_GABRA3 | tcgtgaaagtctgacaaacattTTCCATTAAGAACTTACATTTTTT |
| rs6627588_GABRA3 | TAAACTAGGTGCCACGTCGTGAAAGTCTGACAACCAGAAAACTCTTCCTC |
| rs5970223_GABRA3 | ACTAAACTAGGTGCCACGTCGTGAAAGTCTGACAATCTTCAGCATGCCAACGCT |
| rs7391474_GABRA3 | ACTGACTAAACTAGGTGCCACGTCGTGAAAGTCTGACAAGAGAGACCCAGAGAGAGAAGAAG |
| rs3902802_GABRA3 | cccAACTGACTAAACTAGGTGCCACGTCGTGAAAGTCTGACAAattacCACCCAAGCAGAAATAAGTAAA |
